# Supplementary material for: Peptide barcode of multidrug-resistant strains of Neisseria gonorrhoeae isolated from patients in Thailand
Source: PLoS One. 2023 Aug 3;18(8):e0289308. doi: 10.1371/journal.pone.0289308 (PMC10399818; doi:10.1371/journal.pone.0289308)
Supplement: Attachment — Submitted filename: Response to Reviewers.docx [file pone.0289308.s001.docx]

PONE-D-23-07664

Peptide barcode of multi drug resistant strains of Neisseria gonorrhoeae isolated from patients in Thailand

Reviewer #1: Summary: It is a very interesting and relevant topic. Determining the antibiotic susceptibility of Neisseria gonorrhoeae by mass spectrometry (MS) will help in easy and early detection of antibiotic resistance and hence efficient treatment of patients. Identification of biomarkers of resistance is also very useful.

Overall Impression: The study combines two research questions. First, generating the peptide barcodes for the different groups of antibiotic resistant Neisseria gonorrhoeae, secondly discovering the peptide biomarker of the antibiotic resistance by LC-MS. The results, data and statistical analysis of both the experiments have not been presented adequately in the manuscript.
Major issues:

1. The result part of the MALDI-TOF peptide barcoding, from line number 203, does not clearly provide clear information and data regarding the differences in the peaks of the spectra of the different antibiotic resistance groups mentioned in the study. The Figure 1 is not clear and does not mention the above information. The statistical analysis and Odd's ratio for the same is not reported.

Ans. More informations regarding differences in the Maldi-TOF mass spectra of the different antibiotic resistance groups (Figure 1) and statistical analysis were added in Material and Method section (Line 135-142) and Result section (Line 222-230; Line 236-243).

2. Which test of statistical analysis for the LC-MS test has been done? The figures of the LC-MS tests are not very clear.

Ans. More informations regarding statistical analysis for the LC-MS data by DecyderMS and Mascot were added in Material and Method section (Line 167-174; Line 179-181) and Result section (Line 254-259; Line 261-262). In addition, the peptidomic heatmap, principal component analysis (PCA) and analysis of variance (ANOVA) analysis generated by Metaboanalyst 5.0 were added into Material and Method section (Line 179-181) and Result section (Line 261-262; Line 264-265; Line 267-268).

3. Minor issues: I suggest that the authors hire a copy editor.

Ans. This manuscript was corrected by The Professional editor under the program of Research clinic that supported by Faculty of Allied Health Sciences Thammasat University.

4. The manuscript deals with important topics of detection of antibiotic resistance by Maldi-TOF and discovering peptide biomarker of antibiotic resistance by LC-MS. If the authors can provide the aforementioned details, it will be a valuable contribution to the research in antibiotic resistance.

Ans. As mentioned in Line 254-259. Previous studies have reported the detection of antibiotic resistance bacteria based on peptide barcode differences [18-21]. However, the specific antibiotic resistance peptides on MALDI-TOF mass spectra are difficult to identify due to their low abundance or high molecular weights. LC-MS/MS was therefore used to improve the identification and quantification of potential peptide biomarkers with greater dynamic range, higher resolution, reproducibility and accuracy [23].

Reviewer #2:

1. Novelty statement should be included, as identified 9 peptides are already known for their function in *N. gonorrhoeae*.

Ans. The statement is added (Line 351-357). Peptide biomarkers of antibiotic resistance were associated with resistance enzymes including beta-lactamases, kanR and aminoglycoside modifying enzyme [20]. Some of these resistance peptides were encoded by gene cassettes in the bacterial genomes [21]. However, most of the bacterial peptides were generated by protein degradation [36]. Nine unique peptides were detected in each antibiotic resistant group that originated from nine proteins. These peptides could serve as specific biomarker candidates for AZ, C, CP, CT and CPT-resistant *N. gonorrhoeae.*

2. What are the Inclusion and Exclusion criteria?

Ans. There is no Inclusion and Exclusion criteria for collecting multidrug resistant strains of *Neisseria gonorrhoeae*. We used the antibiotic-resistant *N. gonorrhoeae* samples collected by the Sexually Transmitted Infection Cluster, Bureau of AIDS TB and STIs, Department of Disease Control, Ministry of Public Health, Thailand since January 2019 to December 2021.

3. What are the limitations of the study?

Ans. The limitation of the study is sample size. Although the the collecting time was 2 years (January 2019 to December 2021), only 93 samples were obtained.

4. Why sample size is small?

Ans. Sample size is a limitation of this study. As shown in Material and Method section, only 93 samples were collected during January 2019 to December 2021.
-------------

Reviewer #3:

1. In the abstract and entire document, the name "Neisseria gonorrhoea" should all be typed in italics

Ans. The name "*Neisseria gonorrhoea*" is now typed in italics both in the abstract and entire document.
2. Line 39, insert "of" between prevalence and infection.

Ans. "of" is inserted between prevalence and infection
3. line 56 remove hyphen from the word "de-creased"

Ans. hyphen from the word "de-creased" is removed.
4. Line 70, include year for the citation mentioned.

Ans. The year “2014” is added for the citation mentioned.
5. Line 96, the initial sentence can be re-written as "Sixty-three of the 91 isolates showed resistance to ........"

Ans. This sentence is re-written.
6. Line 112 remove hyphen from the word "performed"

Ans. Hyphen was removed from the word "performed"
7. Change "E test" to "E-test"

Ans. "E test" were changed to "E-test" through the manuscript.
8. In section 2.5, I think is will be appropriate to describe or provide more detail on how the different bacterial groups were pooled together.

Ans. More detail on how the different bacterial groups were pooled together was described in section 2.5 (Line 146-154). The bacterial peptides were extracted from the *N. gonorrhoeae* according to a previous study with slightly modification [11]. Briefly, 5% (v/v) trifluoroacetic acid (TFA) in absolute acetonitrile (ACN) was added to the culture and the suspension was dissolved by gentle vortexing. The samples were dried and resuspended with 0.1% (v/v) formic acid. The Lowry assay was used to determine peptide concentration [12]. Peptides were equally pooled from each bacterium within the same group. Peptidome samples of each bacterial group including azithromycin resistance group (AZ), ciprofloxacin resistance group (C), ciprofloxacin and penicillin resistance group (CP), ciprofloxacin and tetracycline resistance group (CT), ciprofloxacin, penicillin and tetracycline resistance group (CPT) were analyzed by LC-MS.

9. Line 300, use the word "consistent"

Ans. Line 300 is corrected by the Professional editor under the program of Research clinic that supported by Faculty of Allied Health Sciences Thammasat University.

10. IMPORTANT COMMENT: There appears to be a significant missing link in the results presented in this study. The results obtained in the MIC determination using the E-test strip is not shown. The authors should present the result and further correlate with the peptidome obtained for the bacterial isolates. This will provide a further validation of the use of the MALDI-TOF as suggested by the authors.

Ans. The correlation between MICs and peptide barcode is not performed because of broad range of MICs of each bacterial group especially ciprofloxacin and penicillin resistance group (CP), ciprofloxacin and tetracycline resistance group (CT), ciprofloxacin, penicillin and tetracycline resistance group (CPT). Therefore, this study showed that MALDI-TOF peptide barcoding can be used to differentiate between AZ, C, CP, CT and CPT. In addition, LC-MS is used to quantitate and identify potential peptide candidates associated with the multidrug resistant *N. gonorrhoeae.*

Reviewer #4:

1. There are some language and grammatical errors throughout the manuscript text that should be corrected.

Ans. This manuscript was corrected by The Professional editor under the program of Research clinic that supported by Faculty of Allied Health Sciences Thammasat University.

2. Please define the abbreviations at the first mention of them in the text for example, IRB in line 93; PPNG: in line 97.

Ans. Institutional Review Board (IRB) (Line 94) and penicillinase-producing Neisseria *gonorrhoeae* (PPNG) were added (Line 96-97).
3. The data of Table 1 is repeated in the text, so this table could be deleted.

Ans. Table 1 is deleted as suggested.
