# Supplementary material for: Peptide barcode of multidrug-resistant strains of Neisseria gonorrhoeae isolated from patients in Thailand
Source: PLoS One. 2023 Aug 3;18(8):e0289308. doi: 10.1371/journal.pone.0289308 (PMC10399818; doi:10.1371/journal.pone.0289308)
Supplement: Attachment — Submitted filename: Response to Reviewers.docx [file pone.0289308.s002.docx]

PONE-D-23-07664R1

Peptide barcode of multidrug resistant strains of *Neisseria gonorrhoeae* isolated from patients in Thailand

Journal requirements

1. The reference list in line 509-551 has been changed from no 27-37, The reference number 27 was added in line 322 and in line 509 for reference list. The front color of reference no 26 in line 506 was formatted. All References were reviewed and corrected to be complete.

27. Karas M., Glückmann M., Schäfer J. Ionization in Matrix-Assisted Laser Desorption/Ionization: Singly Charged Molecular Ions Are the Lucky Survivors. J. Mass Spectrom. 2000; 35:1–12. doi: 10.1002/(SICI)1096-9888(200001)35:1<1::AID-JMS904>3.0.CO;2-0

2. We upload all new copy of Figures following by PACE.

Response to Reviewers

Reviewer #1: Summary: The issues raised in the previous review have been addressed adequately. The manuscript is also presented in intelligible fashion.

Minor issue:

1. The authors could mention the limitations of the study, the MALDI TOF MS in particular as mentioned in the response to reviewers "However, the specific antibiotic resistance peptides on MALDI-TOF mass spectra are difficult to identify due to their low abundance or high molecular weights"

Answer: The sentences regarding the limitations of the study were added in line 320-328. “Although the application of MALDI-TOF MS for identification of microorganisms has been successful, the single laser MALDI approach produces low ion yield resulting in missing low abundant or hardly ionizable molecules [27]. The low-resolution associated with the linear TOF analyzers and the related mass accuracy might limit the discovery of peptide biomarkers. In addition, MALDI-TOF MS does not allow de novo peptide sequencing in terms of resolution and ability to perform peptide fragmentation and hence identify those specific peptide peaks. According to the limitations of MALDI-TOF MS, mass spectra linked directly to specific antibiotic resistance peptides are difficult to identify. LC-MS/MS was therefore used to identify and quantify potential antimicrobial resistance peptides.”
